# Significance of serum miR‐29a in the occurrence and progression of diabetic nephropathy: A cross‐sectional study

**DOI:** 10.1002/jcla.24210

**Published:** 2021-12-28

**Authors:** Qian Liu, Menglin Wang, Tongdao Xu, Wei Liang, Fumeng Yang

**Affiliations:** ^1^ Department of Laboratory Medicine The Second People’s Hospital of Lianyungang Lianyungang China; ^2^ Department of Laboratory Medicine Lianyungang Hospital Affiliated to Jiangsu University Lianyungang China; ^3^ Department of Laboratory Medicine Suqian First Hospital Suqian China; ^4^ Department of Endocrinology The Second People’s Hospital of Lianyungang Lianyungang China

**Keywords:** diabetic nephropathy, miR‐29a, miRNA, qRT‐PCR, type 2 diabetes mellitus

## Abstract

**Background:**

Diabetic nephropathy (DN), a common microvascular complication of type 2 diabetes mellitus (T2DM), is an important factor causing chronic kidney disease. However, the relationship between miR‐29a and DN remains unknown. Therefore, a cross‐sectional study was conducted to identify a potential molecular biomarker for DN prevention and management by detecting the serum miR‐29a levels.

**Methods:**

The serum miR‐29a levels were measured in 360 subjects (180 T2DM patients and 180 healthy controls) using quantitative reverse transcription PCR (qRT‐PCR), and other conventional indicators were measured and analysed. A binary logistic regression was used to evaluate the DN risk factors; a receiver operating characteristic (ROC) curve was applied to analyse the diagnostic efficacy of miR‐29a for DN, and a Spearman's rank correlation analysis was used to evaluate the correlation between serum miR‐29a and cystatin C.

**Results:**

The serum miR‐29 levels in the T2DM patients were higher than those in the healthy subjects and significantly increased with the progression of DN (*p *< 0.05). Serum miR‐29a and cystatin C are independent predictors of the occurrence of DN. Compared with a single indicator, the combination of serum miR‐29a and cystatin C has better DN diagnostic performance. In addition, the serum miR‐29a levels were positively correlated with cystatin C in the patients with DN (*r* = 0.521, *p *< 0.001).

**Conclusion:**

The expression of serum miR‐29a was significantly associated with the occurrence and progression of DN and is expected to become a potential biomarker for the diagnosis of DN.

## Graphical abstract

Figure (2) ROC curve of serum miR‐29a and cystatin C for patients with DN.

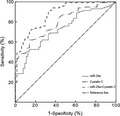

## 1 INTRODUCTION

Type 2 diabetes mellitus (T2DM) is a common metabolic disease that threatens human health, and as it progresses, it often causes a series of microvascular complications.^1^, ^2^, ^3^ Diabetic nephropathy (DN), one of the most common microvascular complications of T2DM, is an important factor causing chronic kidney disease and has been reported to occur in approximately 40% of T2DM patients.^4^ Although the pathogenesis of DN is unclear, its main characteristics include the presence of increased proteinuria, glomerular sclerosis and a decline in the glomerular filtration rate.^5^ Currently, as a noninvasive marker, urinary microalbumin is widely used in the diagnosis, progression and prognosis evaluation of DN. However, the concentration of urinary microalbumin is easily affected by multiple factors, such as urinary tract infection, high blood pressure and vigorous exercise, and its sensitivity and specificity have certain limitations.^6^ In addition, conventional indicators of renal function, such as urea and creatinine, are not sensitive enough for DN screening.^6^ Hence, there is a critical need for sensitive and specific biomarkers to monitor the occurrence and progression of DN, which could be very helpful for clinical interventions to reverse or delay kidney damage in patients with DN.

MicroRNAs (miRNAs) constitute a group of noncoding single‐stranded small RNA molecules with a length of approximately 21–25 nucleotides that can regulate the expression of target genes through the complete or partial complementary binding of the 3’ untranslated region of the target messenger RNA.^7^, ^8^ Strikingly, human serum/plasma contains numerous stably expressed miRNAs and has the biological properties of resistance to RNase activity; thus, miRNAs derived from peripheral blood have the potential to become noninvasive biomarkers of various diseases.^9^, ^10^

Previous studies have indicated that the upregulation of miR‐29a in beta cells induced by glucose may be associated with the progression from impaired glucose tolerance to T2DM, and its expression level in urine was related to albuminuria in patients with T2DM.^11^, ^12^ In addition, recent studies (based on animal models of diabetes) further revealed that miR‐29a plays an important role in the occurrence of glomerular fibrosis, and its potential mechanism may be related to the regulation of the TGF‐β/SMAD3 signalling pathway or the expression of peroxisome proliferator‐activated receptor‐γ (PPAR‐γ) signalling.^13^, ^14^, ^15^ However, most existing reports mainly focus on the potential mechanism of action of miR‐29a in animal models of DN, and systematic analyses of the role of serum miR‐29a in the occurrence and progression of DN in patients have rarely been performed. Therefore, in the present study, the expression levels of serum miR‐29a in T2DM patients, DN patients and nondiabetic healthy individuals were detected using quantitative reverse transcription PCR (qRT‐PCR), and its association with the occurrence and progression of DN was explored to provide a potential molecular biomarker for the prevention and management of DN.

## 2 MATERIALS AND METHODS

### 2.1 Participants

This research was conducted at Lianyungang Hospital Affiliated to Jiangsu University from July 2020 to June 2021. Subjects with T2DM were diagnosed according to the American Diabetes Association criteria as follows: fasting blood glucose (FBG) ≥ 7.0 mmol/L, 2‐hour glucose ≥ 11.1 mmol/L and/or glycated haemoglobin A1c (HbA1c) ≥ 6.5%.^16^ The exclusion criteria were as follows: (1) type 1 diabetes or other types of diabetes; (2) acute infectious disease or inflammation; (3) renal insufficiency not caused by diabetes; (4) nondiabetic proteinuria or albuminuria; (5) various acute or chronic liver, cardiovascular or cerebrovascular diseases; and (6) the use of medication (renin‐angiotensin system inhibitors, SGLT2 inhibitors, GLP‐1 receptor agonists, etc.) or surgery within one month.

In total, 360 participants, including 180 T2DM patients and 180 healthy individuals, were recruited in this research. According to the urinary albumin‐to‐creatinine ratio (UACR), the T2DM patients were divided into the following three groups^17^: (1) normal albuminuria group (NA group, UACR < 30 mg/g, *n* = 64), (2) microalbuminuria group (MA group, 30 mg/g ≤ UACR < 300 mg/g, *n* = 60) and (3) clinical albuminuria group (CP group, UACR ≥ 300 mg/g, *n* = 56). Consistent with the American Diabetes Association guidelines,^18^ the individuals in the MA group and CP group were defined as having DN (UACR ≥ 30 mg/g) in this study. The study protocol was approved by the Ethics Committees of Lianyungang Hospital Affiliated to Jiangsu University (2020X021) in accordance with the Declaration of Helsinki. All participants provided signed informed consent.

### 2.2 Specimen collection and biochemical indicator determination

The subjects fasted for 10–12 h, and blood samples were collected on the following morning. EDTA K_2_ anticoagulated venous blood (2 ml) was used for the testing of HbA1c, and anticoagulant‐free venous blood (5 ml, centrifugal parameters: 1,200 *g* for 10 min) was used for the detection of serum biochemical indicators, such as fasting blood glucose (FBG), total cholesterol (TC), triglycerides (TGs), urea (Urea), creatinine (Crea) and cystatin C. In addition, the remaining serum specimens were stored in a refrigerator at −80℃ for subsequent research (miRNA analysis). Random spot urine specimens (10 ml, centrifugal parameters: 400 *g* for 10 min) were collected for the detection of urinary albumin and Crea, and then, the UACR results were calculated.

The HbA1c levels were measured using commercial reagents on an HLC‐723G8 HbA1c analyser (Tosoh Corporation). The levels of FBG, TC, TGs, Urea, Crea and cystatin C were measured using commercial reagents on an AU5800 biochemical analyser (Beckman Coulter Corporation). The estimated glomerular filtration rate (eGFR) was calculated with the Cockcroft‐Gault equation as follows: eGFR_CG_ = [(140‐age) × weight (kg)]/SCr × 72 × [0.85 if female] and adjusted for a body surface area of 1.73 m^2^.^19^

### 2.3 RNA extraction, reverse transcription and qRT‐PCR analysis

According to the protocol reported by Luo et al.^20^ a one‐step phenol/chloroform purification was used to extract the total RNA from 100 μl of serum. The extracted RNA pellet was dissolved in 20 μl of DEPC water and stored in a refrigerator at −80°C until the subsequent analysis. According to the protocol provided by the reagent manufacturer, the total volume of the reverse transcription reaction was 10 μl and consisted of 2 μl of extracted RNA, 2 μl of 5 × AMV buffer, 0.5 μl of AMV reverse transcriptase (TaKaRa), 3.5 μl of DEPC water, 1 μl of 10 mM dNTPs and 1 μl of stem‐loop RT primer (Applied Biosystems). The reaction conditions for the cDNA synthesis are briefly described as follows: the reaction mixture was incubated at 16°C for 30 min, 42°C for 30 min and 85°C for 5 min and then stored in a refrigerator at 4°C for further analysis.

According to the protocol provided by the reagent manufacturer, qRT‐PCR was performed on a TL988 Real‐Time PCT System (Tianlong Science and Technology Co., Ltd.) using TaqMan probes (Applied Biosystems). Briefly, the total volume of the qRT‐PCR was 20 μl, which consisted of 1 μl of cDNA, 2 μl of 10 × PCR buffer, 0.3 μl of Taq polymerase (TaKaRa), 1.2 μl of 25 mM MgCl_2_, 14.77 μl of DEPC water, 0.4 μl of 10 mM dNTPs and 0.33 μl of TaqMan probe (Applied Biosystems). The reaction conditions for qRT‐PCR were as follows: 95°C for 5 min, followed by 40 cycles (95°C for 15 s and 60°C for 1 min). All samples were equipped with three reaction wells, including a blank control (the sample was replaced with EDPC water). Since conventional reference genes (U6 and 5S rRNA) are unstable in serum samples, they are not suitable as internal references in this experiment. However, since miR2911 (5′‐GGCCGGGGGACGGGCUGGGA‐3′) does not have a mammalian homologue and the measurement of its expression has high repeatability and reproducibility, it was added to each sample during the RNA extraction at a concentration of 10 nmol/L.^21^ The relative concentration of serum miR‐29a was based on the standard concentration of miR2911 and calculated using the comparative Cq method (2^−ΔCq^), where ΔCq was calculated by subtracting the Cq value of miR2911 from the Cq value of miR‐29a. Notably, miR2911 (10 nmol/L) was not subjected to serial dilution and standard curve preparation and was only used as an internal reference to represent the relative expression of miR‐29a (nmol/L). Therefore, the concentration of miR‐29a in this study is not an absolute concentration but a relative concentration obtained from the internal reference (miR2911).

### 2.4 Statistical analyses

The statistical analyses were conducted with SPSS version 19 (IBM Corp.). The Kolmogorov‐Smirnov test was utilized to analyse the normality of the data. The normal variables are expressed as the means and standard deviations, while the nonnormally distributed variables are presented as medians and interquartile ranges (IQRs). To analyse the normally distributed variables, an independent‐samples *t* test was conducted for the comparisons between two groups, a one‐way analysis of variance was conducted for the comparisons of multiple groups, and the LSD test was conducted for the comparisons across multiple groups. To analyse the nonnormally distributed variables, a Mann–Whitney U test was used for the comparisons between two groups, the Kruskal‐Wallis H test was used for the comparisons among multiple groups, and Dunn's post hoc test was used for the pairwise comparisons across multiple groups. To analyse the categorical variables, a chi‐square test was used for the comparisons between two groups. A binary logistic regression analysis was used to assess the independent risk factors of DN, and a receiver operating characteristic (ROC) curve was generated to evaluate the ability of the candidate biomarkers to predict DN. The method of combining multiple variables for a ROC curve analysis is briefly described as follows: the patients (DN patients and controls) were used as the dependent variable, the laboratory test indices (i.e., miR‐29a and cystatin C) were used as the independent variables, a logistic regression equation was constructed, a prediction probability was generated as a new independent variable, and then, this independent variable was further analysed using ROC curves. The correlation of the nonparametric data was evaluated by a Spearman's rank correlation analysis. Two‐tailed *p*‐values less than 0.05 were considered statistically significant.

## 3 RESULTS

### 3.1 Conventional and laboratory characteristics of all subjects

In total, 180 patients with T2DM and 180 healthy controls were included in the present study. The conventional and laboratory indicators of the participants are listed in Table 1. There were no significant differences in age, sex, BMI, smoking status or urea between the T2DM patients and control subjects (*p *> 0.05). The levels of systolic blood pressure (SBP), diastolic blood pressure (DBP), FBG, HbA1c, TC, TGs, UACR, cystatin C and miR‐29a in the T2DM patients were significantly higher than those in the control subjects (*p *< 0.05), while eGFR in the T2DM patients was significantly lower than that in the control subjects (*p *< 0.05).

**TABLE 1:** Conventional and laboratory characteristics of all subjects

Parameters	Control group (*n* = 180)	T2DM group (*n* = 180)	*p*‐Value
Age (years)	53.78 ± 8.22	54.04 ± 7.30	0.7501
Sex (male/female)	98/82	114/66	0.1081
BMI (kg/m^2^)	23.33 ± 1.90	23.63 ± 1.80	0.1202
Smoking status (yes/no)	56/124	71/109	0.1225
Course of disease (years)	‐	10.56 ± 4.27	‐
SBP (mm Hg)	120.30 ± 8.09	130.00 ± 9.70	<0.0001
DBP (mm Hg)	79.10 ± 5.37	83.13 ± 3.68	<0.0001
FBG (mmol/L)	5.13 ± 0.42	9.82 ± 1.94	<0.0001
HbA1c (%)	4.96 ± 0.32	7.31 ± 0.66	<0.0001
TC (mmol/L)	4.74 ± 0.29	5.00 ± 0.36	<0.0001
TGs (mmol/L)	1.07 ± 0.24	1.24 ± 0.33	<0.0001
Urea (mmol/L)	6.56 (5.05, 6.97)	6.75 (5.18, 8.48)	0.0662
Crea (µmol/L)	75 (61, 85)	69 (57, 87)	0.3542
UACR (mg/g)	9.16 (5.61, 12.48)	83.07 (10.18, 482.70)	<0.0001
eGFR (ml/min/1.73m^2^)	103 (99, 108)	99 (93, 105)	<0.0001
Cystatin C (mg/L)	0.76 (0.67, 0.85)	0.91 (0.74, 1.32)	<0.0001
miR‐29a (nmol/L)*	11.30 (9.32, 15.08)	20.66 (12.47, 31.29)	<0.0001

Note

*Indicates that the concentration of miR‐29a is the relative concentration of the internal reference (miR2911).

Abbreviations: BMI, body mass index; Crea, creatinine; DBP, diastolic blood pressure; eGFR, estimated glomerular filtration rate; FBG, fasting blood glucose; HbA1c, glycated haemoglobin A1c; miR‐29a, microRNA‐29a.SBP, systolic blood pressure; T2DM, type 2 diabetes mellitus; TC, total cholesterol; TGs, triglycerides; UACR, urinary albumin‐to‐creatinine ratio; Urea, urea.

### 3.2 Levels of miR‐29a among T2DM patients grouped by UACR

**FIGURE 1:**
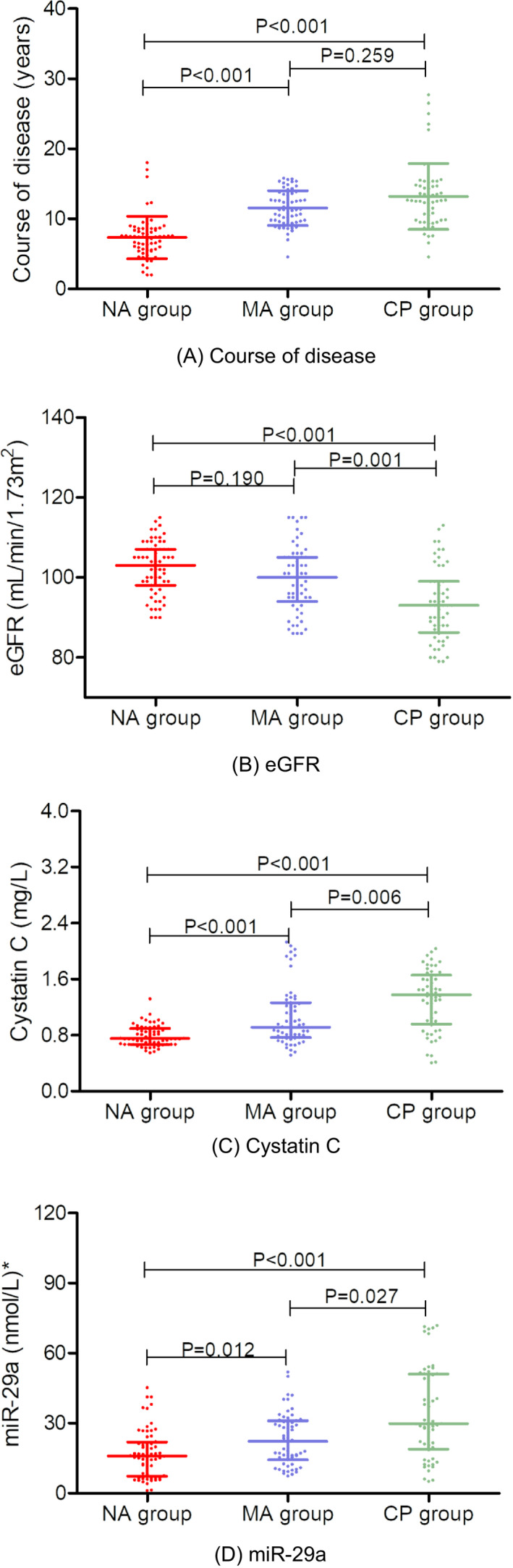
Comparison of the course of disease, eGFR, cystatin C and miR‐29a among patients with T2DM. Scatter plot showing the levels of each indicator among the NA group, MA group and CP group. Error bars in the scatter plot indicate the mean and standard deviation in (A). Error bars in the scatter plot indicate the median and interquartile range in (B), (C) and (D). * indicates that the concentration of miR‐29a is the relative concentration of the internal reference (miR2911)

According to the concentrations of UACR, the T2DM patients were divided into the following groups: NA group, MA group and CP group. There were no significant differences in age, sex, BMI, smoking status, blood pressure (SBP and DBP), FBG, HbA1c, TC or TGs among the T2DM subgroups (*p *> 0.05). The levels of the disease course, miR‐29a, cystatin C and eGFR showed significant differences among the subgroups of T2DM patients. In addition, compared with the NA group, the levels of the disease course, miR‐29a and cystatin C in the DN patients (MA and CP groups) were significantly higher, and the levels of miR‐29a and cystatin C significantly increased with the progression of DN. The detailed information is listed in Table 2 and Figure 1.

**TABLE 2:** Expression levels of biochemical indices among T2DM patients grouped by UACR

Indicators	T2DM			*p*‐Value
	NA group (UACR < 30, *n* = 64)	MA group (30 ≤ UACR < 300, *n* = 60)	CP group (UACR ≥ 300, *n* = 56)	
Age (years)	52.34 ± 6.39	54.77 ± 7.81	55.21 ± 7.46	0.0630
Sex (male/female)	39/25	35/25	40/16	0.3035
BMI (kg/m^2^)	23.30 ± 1.77	23.59 ± 1.76	24.05 ± 1.81	0.0709
Smoking status (yes/no)	19/45	25/35	27/29	0.1066
Course of disease (years)	7.33 ± 3.01	11.53 ± 2.50	13.19 ± 4.70	<0.0001
SBP (mm Hg)	128.80 ± 8.85	131.00 ± 9.98	130.40 ± 10.34	0.4516
DBP (mm Hg)	82.81 ± 3.43	83.50 ± 4.09	83.11 ± 3.53	0.5837
FBG (mmol/L)	9.41 ± 1.67	9.89 ± 1.54	10.20 ± 2.47	0.0732
HbA1c (%)	7.19 ± 0.39	7.31 ± 0.55	7.44 ± 0.95	0.1204
TC (mmol/L)	4.98 ± 0.26	5.00 ± 0.30	5.04 ± 0.49	0.6552
TGs (mmol/L)	1.20 ± 0.26	1.22 ± 0.31	1.29±0.41	0.2822
eGFR (ml/min/1.73m^2^)	103 (98, 107)	100 (94, 105)	93 (86, 99)	<0.0001
Cystatin C (mg/L)	0.76 (0.67, 0.90)	0.92 (0.77, 1.27)	1.38 (0.96, 1.66)	<0.0001
miR−29a (nmol/L)*	16.06 (7.35, 21.94)	22.25 (14.38, 31.00)	29.82 (18.92, 51.05)	<0.0001

Note

*Indicates that the concentration of miR‐29a is the relative concentration of the internal reference (miR2911).

Abbreviations: BMI, body mass index; CP group, clinical proteinuria group; DBP, diastolic blood pressure; eGFR, estimated glomerular filtration rate; FBG, fasting blood glucose; HbA1c, glycated haemoglobin A1c; MA group, microalbuminuria group; miR‐29a, microRNA‐29a.NA group, normal albuminuria group; SBP, systolic blood pressure; T2DM, type 2 diabetes mellitus; TC, total cholesterol; TGs, triglycerides; UACR, urinary albumin‐to‐creatinine ratio.

### 3.3 Independent risk factors of DN

To ensure the accuracy of the binary logistic regression, we conducted a multicollinearity test of all independent variables using SPSS version 19 (IBM Corp.) and finally determined that age, sex, BMI, smoking status, course of disease, SBP, FBG, TGs, cystatin C and miR‐29a were defined as independent variables in this study, while the group allocation (NA, MA and CP groups) was defined as a dependent variable. Subsequently, a binary logistic regression analysis was performed to predict the risk factors for the occurrence of DN. The odds ratios (ORs) of serum miR‐29a and cystatin C for DN were 1.060 (95% CI, 1.018–1.104, *p *= 0.005) and 19.740 (95% CI, 2.526–154.240, *p *= 0.004), respectively.

Meanwhile, we conducted an interaction analysis of the two variables of miR‐29 and cystatin C, and the results showed that there was no interaction between the two variables. Thus, our study indicates that the levels of serum miR‐29a and cystatin are independent risk factors for DN (Table 3).

**TABLE 3:** Binary regression analysis of the risk factors of diabetic nephropathy

Independent variable	B	SE	Wals χ^2^	*p*‐Value	OR (95% CI)
Age	0.052	0.030	2.999	0.083	1.053 (0.993–1.116)
Sex	0861	0.447	3.705	0.054	2.366 (0.984–5.688)
BMI	0.037	0.135	0.074	0.786	1.037 (0.796–1.351)
Smoking status	−0.633	0.443	2.040	0.153	0.531 (0.223–1.266)
Course of disease	0.101	0.071	2.012	0.156	1.106 (0.962–1.271)
SBP	0.031	0.024	1.727	0.189	1.032 (0.985–1.082)
FBG	0.267	0.141	3.577	0.059	1.307 (0.990–1.724)
TGs	0.184	0.835	0.048	0.826	1.202 (0.234–6.173)
Cystatin C	2.983	1.049	8.086	0.004	19.740 (2.526–154.240)
miR−29a	0.058	0.021	7.970	0.005	1.060 (1.018–1.104)
Constant	−15.753	5.451	8.351	0.004	0.000

Note

The results are derived from unadjusted logistic regression analyses.

Abbreviations: B, regression coefficients; BMI, body mass index; CI, confidence interval; FBG, fasting blood glucose; miR‐29a, microRNA‐29a; OR, odds ratio; SBP, systolic blood pressure; SE, standard error; TGs, triglycerides.

### 3.4 ROC analysis of serum miR‐29a and cystatin C levels in patients with DN

**FIGURE 2:**
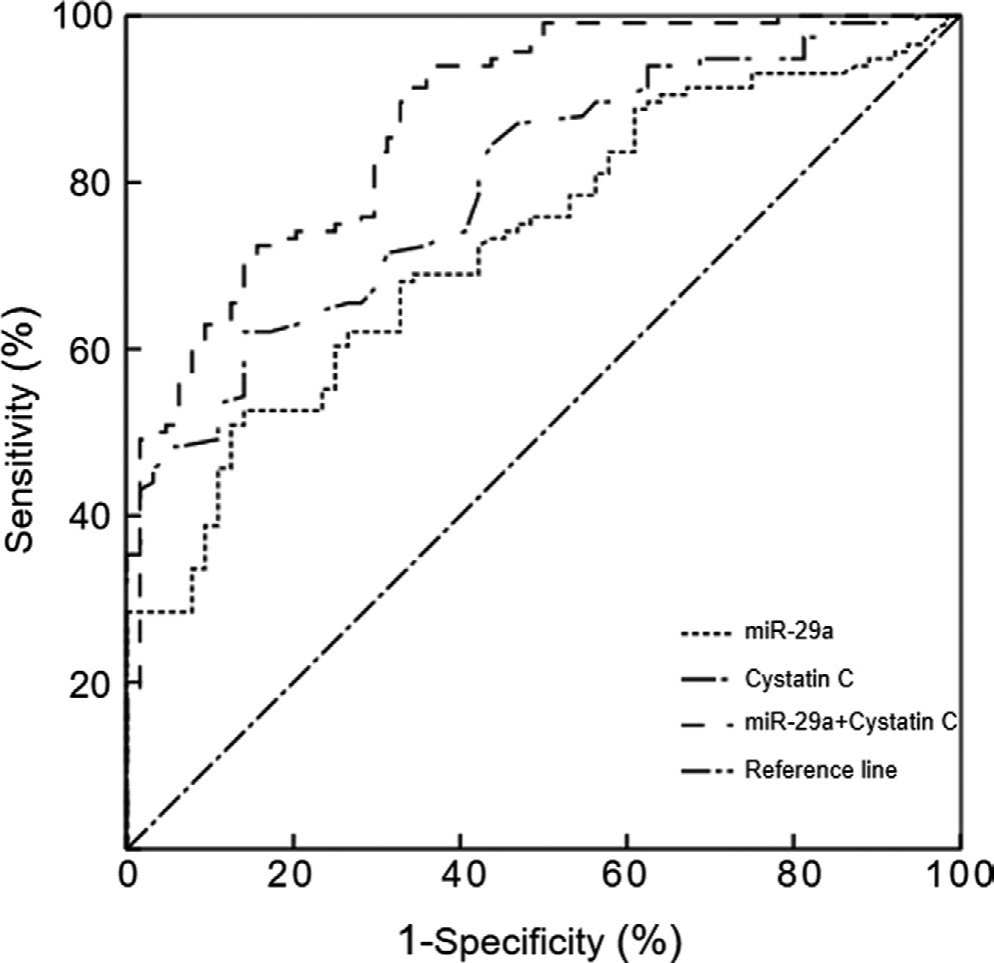
ROC curve of serum miR‐29a and cystatin C in patients with DN. When miR‐29a alone was used, the area under the ROC curve was 0.729 (95% CI: 0.656–0.802; *p *< 0.0001; cut‐off value: 20.66 pmol/L). When cystatin C alone was used, the area under the ROC curve was 0.799 (95% CI: 0.736–0.862; *p *< 0.0001; cut‐off value: 0.95 mg/ml). When the two indicators were combined, the area under the ROC curve was 0.875 (95% CI: 0.824–0.927; *p *< 0.0001)

Referring to the diagnostic criteria for DN, UACR ≥ 30 mg/g was defined as DN.^17^ Based on the above results (miR‐29a and cystatin C are important risk indicators for DN), a ROC curve analysis was performed to evaluate the diagnostic value of miR‐29a and cystatin C for DN. The present study indicates that the combined detection of miR‐29a and cystatin C distinguishes between T2DM patients and DN patients better than the detection of miR‐29a or cystatin C alone (*p *< 0.05, Figure 2). In addition, the Spearman's rank correlation analyses showed that the serum miR‐29a levels were positively correlated with cystatin C in the patients with DN (*r* = 0.521, *p *< 0.001).

## 4 DISCUSSION

Studies have increasingly proven that the expression of circulating miRNAs is dysregulated in various diseases, and miRNAs have been considered promising novel biomarkers for the diagnosis and monitoring of almost all diseases, including DN.^22^, ^23^, ^24^, ^25^ The miR‐29 family comprises miR‐29a, miR‐29b and miR‐29c, of which miR‐29a and miR‐29b are transcribed from chromosome 7q32.3.^26^, ^27^ Moreover, as an important miRNA, miR‐29a is expressed and enriched in pancreatic beta cells in mice and humans, and previous studies have shown that miR‐29a is significantly upregulated in diabetes models.^28^, ^29^, ^30^ However, the expression levels of miR‐29a in the serum of DN patients remain unknown. Therefore, to clarify the level of serum miR‐29a in patients with DN and further evaluate its potential relationship with the occurrence and progression of DN, we used qRT‐PCR to detect the expression levels of miR‐29a in the serum of patients with simple T2DM, patients with DN, and healthy subjects.

In this study, we found that the basic indicators of blood pressure and serum lipids in the T2DM patients were significantly increased compared with those in the healthy subjects. This finding is consistent with those in previous reports, further confirming that changes in blood pressure and blood lipids are involved in the progression of diabetes in some way.^31^, ^32^ This study also revealed that the expression levels of miR‐29a and cystatin C in T2DM patients were significantly higher than those in healthy individuals, while the level of eGFR was significantly decreased. A meta‐analysis conducted by Zhu et al.^33^ confirmed that the expression levels of miR‐29a were significantly upregulated in patients with T2DM and that miR‐29a could become a potential circulating biomarker of T2DM. Another study conducted by Bacci et al.^34^ indicated that the levels of serum cystatin C in a group of patients with HbA1c greater than 7% were significantly higher than those in a group of patients with HbA1c less than 7%. In addition, Bacci et al. found that the levels of eGFR in the high‐HbA1c group (HbA1c ≥ 7%) were significantly lower than those in the low‐glycated haemoglobin group (HbA1c < 7%). The above‐mentioned studies are consistent with ours and fully confirm that serum miR‐29a, cystatin C and eGFR are related to the progression of T2DM.

Based on the levels of UACR, the T2DM patients were divided into three groups (the NA, MA and CP groups). Our study refers to the diagnostic guidelines for DN, and the latter two groups of subjects were defined as patients with DN.^18^ Compared with the patients with simple diabetes, the course of disease in the DN patients was significantly increased. We also found that the expression level of miR‐29a in the serum of patients with DN was significantly higher than that in patients with simple diabetes, and the expression level of miR‐29a gradually increased with the progression of DN. In addition, we observed that the level of cystatin C in the serum showed a corresponding changing trend. Subsequently, a binary logistic regression was performed to analyse the independent risk factors for DN, and the results clarified that miR‐29a and cystatin C are important risk factors for predicting the occurrence of DN. According to the above findings, we conducted in‐depth research. In this study, based on the differences in miR‐29a and cystatin C expression in subgroups of T2DM subjects, their diagnostic efficacy for DN was determined by using ROC curves. We found that serum miR‐29a and cystatin C have important clinical value in differentiating DN from simple diabetes. Moreover, the combined detection of miR‐29a and cystatin C can further improve its diagnostic efficacy for DN. In the present study, we also found that the serum miR‐29a levels were positively correlated with cystatin C in the DN patients. A study conducted by Chien et al.^35^ indicated that the levels of miR‐29a were significantly upregulated in patients with overt proteinuria compared with those in patients with microalbuminuria and/or overt proteinuria. These authors also found that DN progressors showed significantly increased expression of miR‐29a in comparison with nonprogressors. Another study conducted by Sun et al.^36^ confirmed the key role of macrophage‐related inflammation in the progression of miR‐29a‐induced diabetes. The above‐mentioned studies are highly consistent with our results, which fully demonstrate that the levels of miR‐29a in DN patients are significantly upregulated, indicating that DN‐related miRNAs have potential clinical value in monitoring and preventing diseases.

There are several limitations in our study that need to be mentioned. First, this study was a single‐centre study, and the characteristics of the subjects were not sufficiently diverse, thereby restricting the generalizability of our findings. Second, because this study was a cross‐sectional study, follow‐up of the subjects was not completed; thus, the relationship between miR‐29a and the prognosis of DN was not clarified. Third, although the concentrations of serum miR‐29a in the occurrence and progression of DN were investigated systematically in this study, the molecular mechanism of miR‐29a in the development of DN is still unclear. In our future research, we need to carry out a multicentre case‐control study and establish an animal model of DN to clarify the pathological mechanism of the involvement of miR‐29a in DN and increase its clinical value.

## 5 CONCLUSION

In conclusion, the expression of serum miR‐29a was significantly associated with the occurrence and progression of DN and is expected to become a potential biomarker for the diagnosis of DN. The underlying mechanism by which serum miR‐29a correlates with DN needs to be explored in depth in basic research.

## CONFLICT OF INTEREST

All authors declare that they have no conflicts of interest.

## AUTHOR CONTRIBUTIONS

QL and FMY conceived and designed the experiments. QL, MLW and TDX performed the experiments. MLW and WL analysed the data. QL and FMY wrote the first draft of the manuscript. All authors accept responsibility for the entire content of this submitted manuscript and approve its submission.

## Data Availability

The data are available upon request from the corresponding author (Fumeng Yang).
